# Emerging challenges in antimicrobial resistance: implications for pathogenic microorganisms, novel antibiotics, and their impact on sustainability

**DOI:** 10.3389/fmicb.2024.1403168

**Published:** 2024-04-29

**Authors:** Shikha Sharma, Abhishek Chauhan, Anuj Ranjan, Darin Mansor Mathkor, Shafiul Haque, Seema Ramniwas, Hardeep Singh Tuli, Tanu Jindal, Vikas Yadav

**Affiliations:** ^1^Amity Institute of Environmental Sciences, Amity University, Noida, Uttar Pradesh, India; ^2^Amity Institute of Environmental Toxicology, Safety and Management, Amity University, Noida, Uttar Pradesh, India; ^3^Academy of Biology and Biotechnology, Southern Federal University, Rostov-on-Don, Russia; ^4^Research and Scientific Studies Unit, College of Nursing and Health Sciences, Jazan University, Jazan, Saudi Arabia; ^5^Gilbert and Rose-Marie Chagoury School of Medicine, Lebanese American University, Beirut, Lebanon; ^6^University Centre for Research & Development, University Institute of Pharmaceutical Sciences, Chandigarh University, Mohali, Punjab, India; ^7^Department of Bio-Sciences and Technology, Maharishi Markandeshwar Engineering College, Maharishi Markandeshwar (Deemed to Be University), Ambala, India; ^8^Department of Translational Medicine, Clinical Research Centre, Skåne University Hospital, Lund University, Malmö, Sweden

**Keywords:** antibiotic resistance, mechanisms of resistance, gene transfer, novel antibiotic, sustainability, sustainable development goals

## Abstract

Overuse of antibiotics is accelerating the antimicrobial resistance among pathogenic microbes which is a growing public health challenge at the global level. Higher resistance causes severe infections, high complications, longer stays at hospitals and even increased mortality rates. Antimicrobial resistance (AMR) has a significant impact on national economies and their health systems, as it affects the productivity of patients or caregivers due to prolonged hospital stays with high economic costs. The main factor of AMR includes improper and excessive use of antimicrobials; lack of access to clean water, sanitation, and hygiene for humans and animals; poor infection prevention and control measures in hospitals; poor access to medicines and vaccines; lack of awareness and knowledge; and irregularities with legislation. AMR represents a global public health problem, for which epidemiological surveillance systems have been established, aiming to promote collaborations directed at the well-being of human and animal health and the balance of the ecosystem. MDR bacteria such as *E. coli*, *Staphylococcus aureus*, *Pseudomonas aeruginosa*, *Enterococcus* spp., *Acinetobacter* spp., and *Klebsiella pneumonia* can even cause death. These microorganisms use a variety of antibiotic resistance mechanisms, such as the development of drug-deactivating targets, alterations in antibiotic targets, or a decrease in intracellular antibiotic concentration, to render themselves resistant to numerous antibiotics. In context, the United Nations issued the Sustainable Development Goals (SDGs) in 2015 to serve as a worldwide blueprint for a better, more equal, and more sustainable existence on our planet. The SDGs place antimicrobial resistance (AMR) in the context of global public health and socioeconomic issues; also, the continued growth of AMR may hinder the achievement of numerous SDGs. In this review, we discuss the role of environmental pollution in the rise of AMR, different mechanisms underlying the antibiotic resistance, the threats posed by pathogenic microbes, novel antibiotics, strategies such as One Health to combat AMR, and the impact of resistance on sustainability and sustainable development goals.

## 1 Introduction

The spread, persistence, and prevalence of antibiotic-resistant bacteria (ARB) and antibiotic-resistant genes (ARG) in the environment because of excessive and improper use of human and veterinary antibiotics is a global concern and issue. It is well acknowledged that antibiotic resistance poses a serious threat to human health on a global scale. In addition, multidrug-resistant (MDR) organisms have been frequently reported in community settings apart from hospitals, indicating that reservoirs of ARB exist elsewhere outside hospitals such as in water or aquatic environment (Munita and Arias, 2016), soil (Seyoum et al., 2021), foods (Shen et al., 2022) and air (Zhou et al., 2021). As bacteria continue to proliferate and evolve new forms of resistance to antimicrobial agents, some emerging community-associated illnesses may impede the progress of bacterial infections and diseases. The ARB and ARGs have been found in a variety of environmental compartments, including wastewater treatment plants, aquaculture farms, hospital wastewater, confined animal feeding operations, and surface and groundwater (Lamba et al., 2017; Chen et al., 2018; Manaia et al., 2018).

According to Lupo et al. (2012), one of the most dangerous health issues currently facing the world is bacterial antibiotic resistance evolution & its dissemination and appearance. Antibiotic resistance poses a severe concern to human health such as prolonged illness and increased death since it can spread from environmental compartments to drinking water sources as a main route of exposure. Freshwater in particular acts as a reactor in which novel resistances develop and become more prevalent. Numerous investigations found ARB and ARGs in untreated drinking water sources such as wells, groundwater, rivers, and lakes as well as in tap and bottled water (Sanganyado and Gwenzi, 2019; Kaiser et al., 2022; Li et al., 2023). Hydrological processes including runoff, infiltration, leachates, and groundwater recharge, as well as anthropogenic activity like wastewater discharge, etc. are what cause Antibiotic Resistance to spread from the various reservoirs into drinking water sources. The existence of resistance determinants in environmental and soil bacteria does not directly pose a threat to human health. However, when these determinants are mobilized to new hosts and expressed in different contexts, such as their transfer to plasmids and integrons in pathogenic bacteria, it can lead to a significant problem with widespread implications (Peterson and Kaur, 2018). The types and dosages of antibiotics administered to the animals are reflected in the antimicrobial resistance patterns seen in the animals (Reygaert, 2018). The direct oral route (which includes eating meat and ingesting faeces in contaminated water or food) is the most prevalent way for antibiotic resistance to spread from animals to humans. Direct human contact with the animals is another typical route (Wegener, 2012). Pharmaceutical industrial waste, human and animal faeces can release antibiotics and antibiotic resistance into the environment. Antibiotic-related water contamination is an environmental danger that must be addressed by the entire population. Antibiotics are found extensively in water and are eventually absorbed or digested by aquatic species. When antimicrobials are utilised in farmed fish production, AMR genes may reach the food chain and, eventually, the human body via seafood intake. High amounts of antibiotics in cattle manure can infiltrate the soil and water environment in a variety of ways, polluting the ecosystem. Residual antibiotics can enter the soil by animal dung and urine fertilisation and accumulate there, affecting soil fertility, crop chlorophyll production, enzyme release, and root development. Antibiotic residues also have an impact on the structure and activity of the soil microbial community, as well as the development and dissemination of antibiotic-resistant bacteria and resistance genes (Tian et al., 2021). Other sources of antibiotic contamination include hospitals, where antibiotics are commonly used to treat bacterial infections. Improper treatment of hospital wastewater discharges leads to the diffusion of antibiotics into the soil, and its reuse in crop irrigation of economically significant plants such as rice and wheat leads to antibiotic contamination (Zhu et al., 2019; Barathe et al., 2024).

Second-line antibiotics are more readily available in developing nations, which reduces the need for next-generation medicines and increases morbidity and death from illnesses that are resistant to antibiotics (Shiadeh et al., 2019). As per the World Health Organization (2014), one of the most serious three public health problems of the twenty-first century, is antibiotic resistance. Antibiotic-resistant illnesses kill at least 0.7 million people worldwide each year, and within 30 years, it is anticipated that the number would rise to 1.0 million which could significantly outnumber cancer-related fatalities (Lerminiaux and Cameron, 2019).

Modern medicine was also revolutionised by the discovery, widespread use, and commercialization of antimicrobial drugs for the treatment of infections, which also altered the therapeutic paradigm (Munita and Arias, 2016). Even though antimicrobials are crucial clinical tools for curing and preventing infectious diseases, resistance is still erupting, diversifying, and spreading quickly. The spread of resistant organisms, treatment failures, elevated mortality rates, the potential for undetected low levels of resistance, and the associated burden on healthcare expenses make the increase in microbial resistance a serious problem. Since antibiotic-resistant germs cannot be destroyed or inhibited in their growth, they appear to be more virulent. To find additional targets and develop the next generation of powerful antimicrobials, numerous efforts have been made to overcome the resistance (Murugaiyan et al., 2022).

Mutations are one of the causes of antibiotic resistance development (Urban-Chmiel et al., 2022). Mutations occurring in already-existing genes of the bacterial chromosome that are subsequently positively chosen by environmental pressures, drive the evolution of all known antibiotic resistance mechanisms acquired by opportunistic and pathogenic bacteria. Two concurrent evolutionary factors are involved in the long-term preservation of antibiotic resistance genes in bacterial communities: selection favouring resistance phenotypes and selection reducing the fitness costs associated with carrying resistance genes (Bengtsson-Palme et al., 2018). Based on antimicrobial processes such as suppression of bacterial metabolic pathways, inhibition of protein synthesis, inhibition of nucleic acid synthesis, and inhibition of cell membrane depolarization, antibiotics can be categorised into several classes. Epigenetics may have a significant impact on how bacteria become resistant to antibiotics, according to many recent reports. The rapid emergence of drug resistance in bacteria can be explained by the inherent heterogeneity and temporary nature of epigenetic inheritance. Adenine and Cytosine Methylation can modulate mutation rates in bacterial genomes, modifying antibiotic susceptibility (Ghosh et al., 2020). The two types of antibiotic resistance mechanisms are acquired/genetic (chromosomal mutations and extra-chromosomal plasmid) and natural/intrinsic (Munita and Arias, 2016). Resistance mutations commonly arise in genes which encode important operations; as a result, these mutations are usually harmful in the absence of medications (Durão et al., 2018).

Horizontal Gene Transfer (HGT) gives infectious bacteria a path by which an antibiotic resistance gene (ARG) might cause an outbreak by spreading resistance across numerous unrelated bacteria (Lerminiaux and Cameron, 2019), such as sulfonamide resistance genes (*sul1*, *sul2* and *sul3*), encoding a dihydropteroate synthase (DHPS), play a crucial role in bacterial resistance to sulfonamides by causing the bacteria to develop a low affinity for sulfonamides. These genes are found on mobilised plasmids with transposons and a host range, which causes the bacteria to acquire numerous sulphonamide-co-selected antibiotic resistances (Adekanmbi et al., 2020). The horizontal acquisition of ARGs leads to the diversification of genomes and generates a potential for when bacteria are subjected to strong selective pressures such as the presence of antimicrobials. HGT can produce the genes needed for survival more quickly than spontaneous mutations (Lerminiaux and Cameron, 2019).

According to guidelines given by the WHO in the year 2021 and other members of the AMR Tripartite, AMR might have a substantial impact on a variety of the United Nations Sustainable Development Goals (SDGs) such as AMR raises treatment costs, many nations may be unable to afford the universal health care (Berkner et al., 2014). Addressing AMR is critical to accomplishing the SDGs. Many of the goals (e.g., greater access to clean water and sanitation, sustainable consumption, and production, such as more sustainable food production, and proper use of antimicrobials in people and animals) will contribute to addressing AMR. At the same time, growing AMR levels will make it more difficult to meet health, poverty reduction, food security, and economic growth goals.

**Figure 1:**
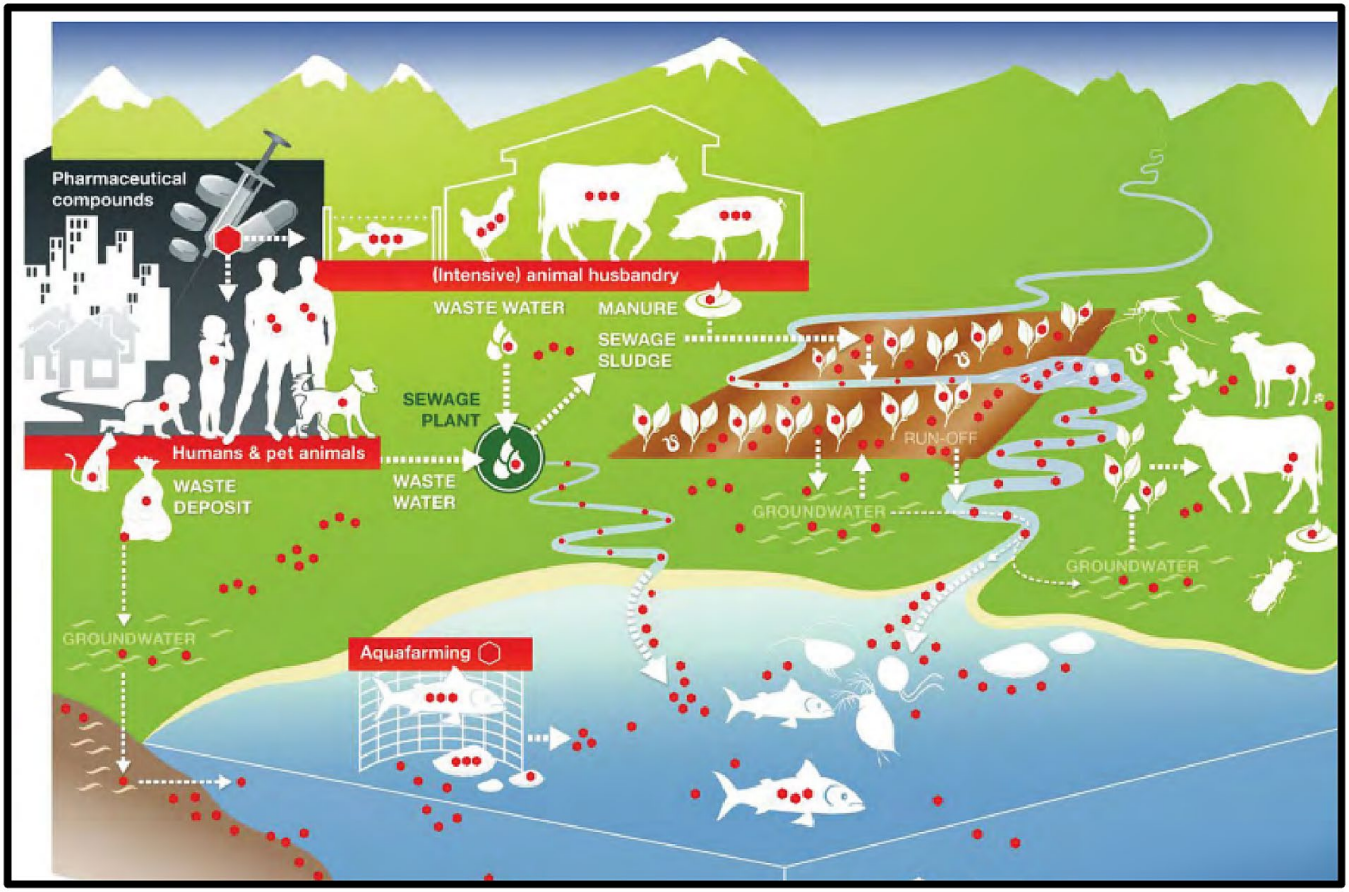
Spread of Antibiotic Resistance in the ecosystem [Adopted from Berkner et al. (2014)].

The continued rise of AMR, for example, may hinder the achievement of Sustainable Development Goals 1 (No Poverty) and 2 (Zero Hunger). Food production is expected to increase by 50–70% between 2010 and 2030 due to increasing global population, economic growth, and changes in consumption habits; at the same time, the use of antimicrobials in food production (e.g., meat, milk, eggs) is expected to increase by a similar margin (Laxminarayan et al., 2016). Major reports have already demonstrated this: while global antibiotic consumption in human medicine increased by 10%, consumption of these drugs in animal husbandry increased by nearly 180% in the “BRICS” conglomerate countries (Brazil, Russia, India, China, and South Africa), with continued rising trends expected till 2030 (Van Boeckel et al., 2017), which will lead to the spread of AMR across the ecosystem (Figure 1).

In this review, we have explored the contemporary understanding and insights into the mechanisms driving antibiotic resistance among diversified bacteria, while also addressing the imminent threats posed by antibiotic resistance. Additionally, it aims to examine emerging novel antibiotics and evaluate the potential impacts of antibiotic resistance on achieving Sustainable Development Goals.

## 2 The mechanisms underlying antibiotic resistance

The organisms cannot be destroyed or stopped from growing, antibiotic-resistant strains seem to be more virulent. Both bacterial uptake of foreign DNA and changes in a bacterium’s pre-existing genome can result in antibiotic resistance. In human or animals receiving antibiotic treatment, mutations happen easily and become fixed (Larsson and Flach, 2022). Even more frequent mutations result in genomic rearrangements (insertions, deletions, duplications, and inversions), which can hasten the development of antibiotic resistance. Physiology, genetics, antibiotic-bacterium interactions (e.g., an antibiotic itself can affect mutation rate, or different resistance mutations can be selected at different antibiotic doses) and the environment to which bacteria have been exposed in the past and present all have an impact on how quickly AR mutants emerge in bacteria (Harmand et al., 2017; Durão et al., 2018).

**Figure 2:**
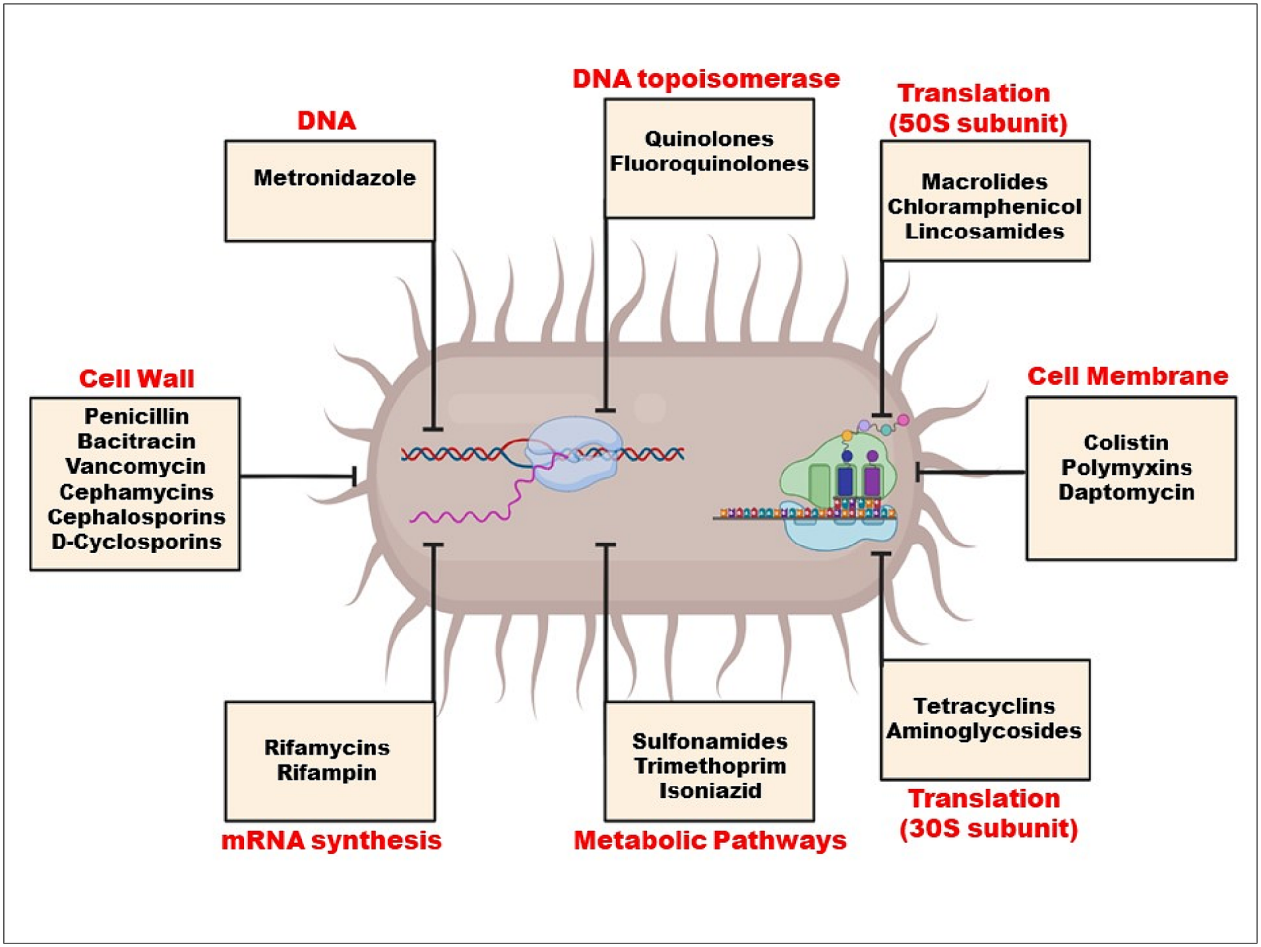
Mechanism of action of antibiotics.

Selection pressure on resistance genes is created by the rise in antibiotic concentrations in the environment, especially in human waste streams where resistance genes are produced and mixed with antibiotics and other biocidal chemicals (Martínez et al., 2017). When environmental bacteria are exposed to this mixture, horizontal gene transfer (HGT) events are triggered (Michaelis and Grohmann, 2023), which spread genetic resistance components throughout various strains and species, increasing the microorganism’s population and allowing it to colonise new niches and hosts. By using HGT processes, all of the genes from the extrinsic resistome can be transferred to pathogenic and non-pathogenic bacteria from a variety of habitats, including people and other animals as well as other ecosystems (Bello-López et al., 2019). When drugs are blocked from entering cells due to the absence of a target, such as a cell wall, intrinsic resistance develops (Figure 2). These bacteria include *Klebsiella*, *Pseudomonas aeruginosa*, and *E. coli*.

**Figure 3:**
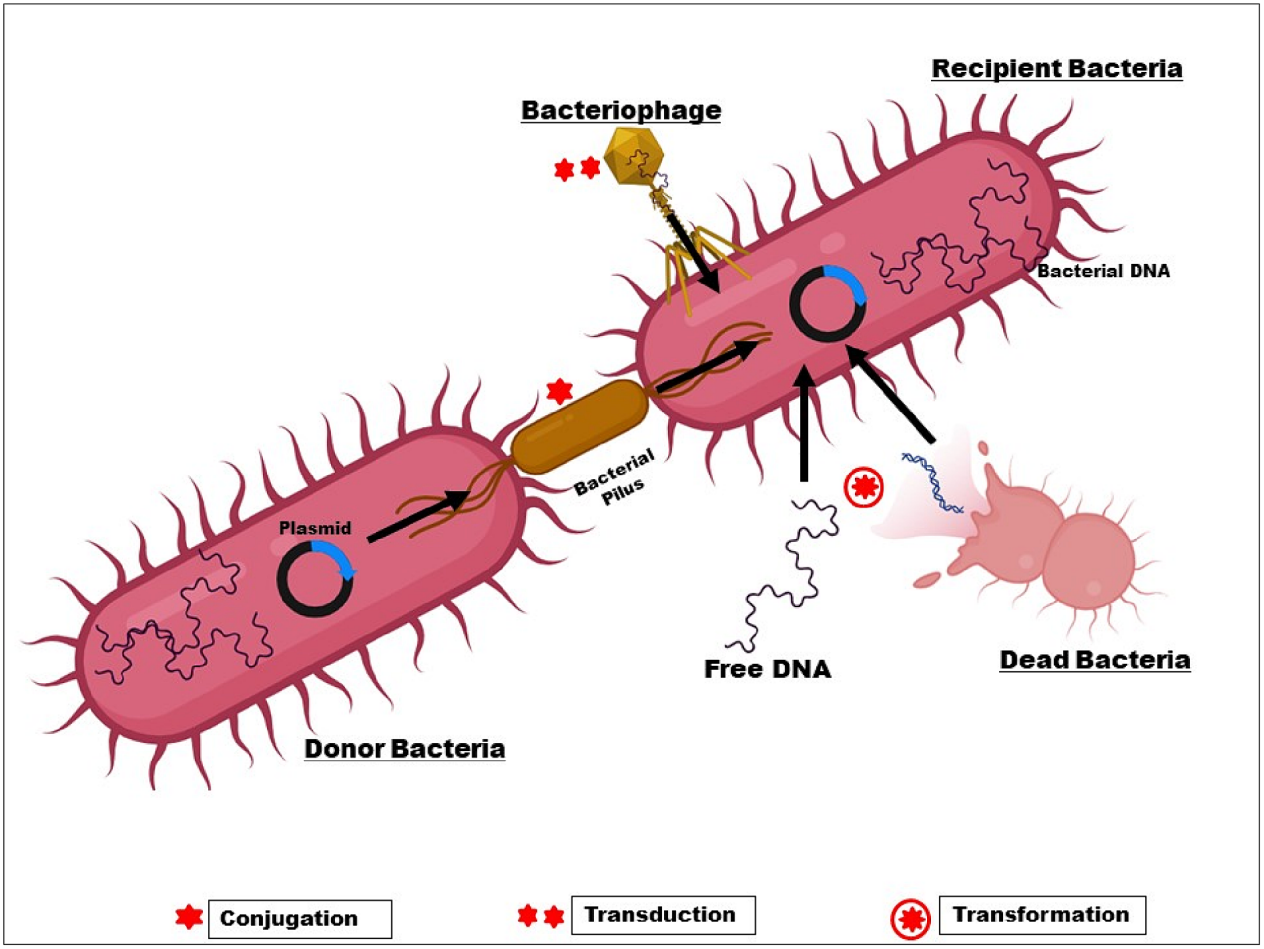
Transfer of r-gene from one bacterium to another.

On the other hand, changes in bacterial genetic makeup result in decreased antibiotic sensitivity and higher acquired resistance (Uddin et al., 2021). Plasmid-mediated medication resistance is common in clinical settings. Genetic material found outside of chromosomes can multiply on its own in the cytoplasm. Gene resistance (r-genes) is present in these plasmids and is easily transfected from one plasmid to another. r-genes are easily transposable between R-plasmids and chromosomes. The three main agents that promote HGT through conjugation, transduction, and natural transformation are plasmids, bacteriophages, and extracellular DNA (Figure 3).

Antibiotics can induce conjugation, which is important in clinical settings where antibiotic usage is common. In the *Enterobacteriaceae*, *Pseudomonas*, and *Acinetobacter*, B-lactam resistance genes are frequently found on plasmids and spread through inter-and intra-species conjugation (Martin et al., 2017; Phan et al., 2017; Gorrie et al., 2018). Globally, the plasmid pCT disseminated the blaCTX-M-14 to both animals and people on various continents. The widespread and quick spread of ESBLs suggests that these genes are instantly functional in newly formed host cells after HGT.

From the perspective of evolution, bacteria use two main genetic strategies to adapt to the antibiotic “attack”: (i) mutations in gene(s) frequently linked to the compound’s mechanism of action; and (ii) acquisition of foreign DNA through horizontal gene transfer (HGT) coding for resistance determinants (Munita and Arias, 2016).

Limiting a drug’s absorption, altering a drug target, inactivating a drug, and active drug efflux are the basic mechanisms of resistance. Both endogenous to the bacteria and acquired from other microorganisms can be responsible for these mechanisms (Reygaert, 2018) (Table 1).

**Table 1:** List of antibiotics used for different bacteria and their resistance mechanism.

S.No	Organism/strain	Resistance mechanism	Antibiotics used	References
1	*Enterococci* spp.	Acquiring B-lactamases or having PBP4/5 mutations	Penicillin	Hollenbeck and Rice (2012)
2	*Enterobacteriaceae, Klebsiella pneumoniae*, *Pseudomonas aeruginosa* and *Acinetobacter* baumannii	Porin mutations, efflux pumps, and enzyme production	Carbepenams	Suay-García and Pérez-Gracia (2021) and Codjoe and Donkor (2017)
3	*Enterococcus* spp.	Altering the process for the production of peptidoglycans, specifically by changing D-Alanine-D-Alanine (D-Ala-D-Ala) to either D-Alanine-D-Lactate (D-Ala-D-Lac) or D-Alanine-D-Serine (D-Ala-D-Ser).	Glycopeptides	Ahmed and Baptiste (2018)
4	*Pseudomonas aeruginosa*	driven by the aminoglycoside modifying enzymes (AME) like byaac(6′)-I, aac(6′)-II, ant(2″)-I, and aph(3′)-VI,	Aminoglycosides	Teixeira et al. (2016)
5	*Pseudomonas aeruginosa*	Limited permeability of the outer membrane, efflux systems that remove antibiotics from the cell, the development of B-lactamases and other antibiotic-inactivating enzymes, mutations, or horizontal gene transfer acquisition of resistance genes.	Sulfonamides	Pang et al. (2019) and Breidenstein et al. (2011)
		Antibiotic cleavage by β-lactamse enzymes, antibiotic expulsion by chromosomally encoded efflux mechanisms and reduced drug uptake owing to loss of outer membrane porin proteins.	Cephalosporins	Poole (2011)
		Antibiotic inactivation by Chloramphenicol acetyl transferase (CAT)	Chloramphenicol	Kapoor et al. (2017)
		Production of proteins that bind to the ribosome and alter the conformation of the active site	Tetracycline	Kapoor et al. (2017)
6	*Escherichia coli, Klebsiella pneumonia, Staphylococcus aureus*	Errors in the replication of the genes encoding the GyrA subunits of DNA gyrase and ParC of topoisomerase IV Overexpression of efflux pumps from the resistance-nodulation-cell division, Reduction of the membrane permeability by down regulation of extra-membrane proteins	Quinolones	Solano-Gálvez et al. (2020)
	*Enterobacteriaceae species, Pseudomonas aeruginosa, Staphylococci, Streptococcus pneumoniae, Enterococcus faecalis* and *Listeria monocytogenes*	Mutations in both target enzymes DNA gyrase in Gram-negative bacteria & topoisomerase IV in Gram-positive bacteria and increased expression of chromosomal gene leading to increased efflux of the fluoroquinolones.	Fluroquinolones	Yadav and Talwar (2019)
9	*Enterococci* spp., & *Staphylococcus aureus*	Mutations in the domain V region of 23S rRNA genes; acquisition of the ribosomal methyl-transferase gene *cfr*; and mutations in *rpl*D, and *rpl*C genes that encode 50S ribosomal proteins L4 and L3 respectively	Oxazolidinones	Stefani et al. (2010)
11	*Klebsiella pneumoniae*	Presence of the SHV-1 penicillinase in their chromosome, chromosomal mutations, and from acquisition of AMR genes via HGT, mainly via large conjugative plasmids.	Ampicillins	Wyres and Holt (2018)

### 2.1 Limited drug uptake

Microbes may develop defence mechanisms that prevent an antimicrobial medicine from building up, which subsequently prevents the antibiotic from reaching its intended cellular target. Certain types of chemicals are blocked by the shape and functions of the lipopolysaccharide layer in gram-negative bacteria. Because of this, certain bacteria have an intrinsic resistance to classes of powerful antimicrobial medicines (Reygaert, 2018).

### 2.2 Drug target modification

Target site modifications frequently result from a bacterial gene’s chromosomal spontaneous mutation. Minor changes to the target molecule can have a significant impact on antibiotic binding since antibiotic interactions with targets are typically extremely selective (Kapoor et al., 2017). Species that manufacture aminoglycosides, like *Streptomyces* spp., have this resistance mechanism, which gives them inherent resistance to the antibiotics they make. Enzymatic inactivation, however, is the most typical aminoglycoside resistance mechanism (Laws et al., 2019). Drugs that impact protein synthesis, such as macrolides, tetracycline, chloramphenicol, and aminoglycosides, become resistant when the 30S or 50S subunits of the ribosome are altered (Anandabaskar, 2021). While chloramphenicol, macrolides, lincosamides, and streptogramin B bind to the 50S ribosomal subunit to inhibit protein synthesis, AGs (aminoglycoside) bind to the 30S ribosomal subunit (Haider et al., 2022).

### 2.3 Drug inactivation

There are two basic ways that bacteria inactivate drugs: by degrading the medicine, or by adding a chemical group to the drug (Noor et al., 2021). The bacterial enzymes have been demonstrated to add chemical groups to antibiotic molecules’ susceptible regions, preventing the antibiotic from attaching to its intended target (Ali et al., 2018). Hydroxyl and amide groups within an antibiotic’s structure are easily altered by hydrolysis (Mehta et al., 2016). For instance, *Staphylococcus aureus* has demonstrated penicillin resistance, which is caused by the blaZ gene, which produces the B-lactamase enzyme; by inhibiting the cross-links that make up the bacterial cell wall from forming, B-lactam antibiotics hinder the manufacture of the wall and weaken it, preventing bacterial growth (Monistero et al., 2020).

### 2.4 Active drug efflux

Reduced permeability and/or antibiotic efflux are two key mechanisms of antibiotic resistance in clinical strains. Gram-negative bacteria rely on the presence of their outer membrane, which acts as a permeability barrier, offering a pre-existing defence mechanism against hydrophilic antibiotics and other antimicrobial drugs like vancomycin. Thus, maintaining decreased permeability is vital for Gram-negative bacteria (Peterson and Kaur, 2018). Active efflux out of the cell frequently leads to resistance to B-lactams, tetracyclines, and fluoroquinolones, and it is rather typical for a single efflux pump to be able to transport a variety of antimicrobials. Except for polymyxin, all antibiotic classes are prone to activating efflux mechanisms. Efflux pumps can confer significant degrees of resistance to formerly therapeutically effective antibiotics when they are overexpressed. Many efflux pumps, also referred to as multidrug resistance (MDR) efflux pumps, transport a large variety of structurally diverse substrates, in contrast to some efflux pumps that have a tight substrate specificity such as Tet pumps (Blair et al., 2015).

## 3 Alarming rise of antimicrobial resistance in pathogenic bacteria: a cause for concern

The formulation of thousands of tonnes of antibiotics has been spurred by population expansion, rising prosperity, and inappropriate use, and it is anticipated that production will continue to rise in the future years. A growing amount of evidence over the past few decades has demonstrated that antibiotics entering the environment can potentially have negative impacts on humans and non-target creatures (Christou et al., 2017). Antibiotics are released into the environment because of a variety of human actions, including the usage of veterinary drugs and the direct dumping of unused or expired prescriptions.

### 3.1 *Enterococci* spp.

*Enterococci’s* clinical importance is mostly connected to their antibiotic resistance, which promotes colonisation and infection, particularly in hospitalised patients. Between 2001 and 2015, *Enterococcus faecalis* species had a lower rate of resistance than *Enterococcus faecium*. However, both species’ rates of resistance significantly rose during this time. In comparison to general cases (hospitalised or outpatients without a specific underlying disorder), complicated cases (patients with underlying debilitating disorders) had higher mean resistance rates for vancomycin, gentamicin, ciprofloxacin, erythromycin, nitrofurantoin, chloramphenicol, trimethoprim-sulfametoxazol, imipenem, and teicoplanin. Except for penicillin and ampicillin, difficult cases had greater rates of antibiotic resistance than general patients (Asadollahi et al., 2018). *Enterococci species* (*E. faecalis*, *E. faecium*, and *E. casseliflavus*) found in 150 wild birds were tested for antimicrobial susceptibility. Antibiotics tetracycline, erythromycin, chloramphenicol, gentamicin, and streptomycin all showed high rates of resistance, while ampicillin and linezolid had low rates of resistance (Yahia et al., 2018). To characterize the virulence factors and antibiotic resistance profiles in enterococci taken from various clinical sources in the northwest of Iran, Jahansepas et al. (2018) conducted a study. From September 2014 to July 2015, 160 *enterococcal* clinical isolates were recovered from various wards of University Teaching Hospitals and identification was done by biochemical assay. 125 (78.12%) and 35 (21.88%) of the 160 *enterococcal* isolates were found to be *Enterococcus faecalis* and *Enterococcus faecium*, respectively. Resistance to rifampicin (76.25%) was the most prevalent pattern of antibiotic non-susceptibility, followed by erythromycin (73.12%). Vancomycin resistance was discovered in 30 isolates (11 *E. faecalis* and 19 *E. faecium*), with a minimum inhibitory concentration of >256 mg/mL (Jahansepas et al., 2018; Chauhan and Jindal, 2020).

The prevalence of vancomycin-resistant *E. faecium* in bloodstream infections is notably high, especially in Eastern Mediterranean nations, according to a 2019 meta-analysis study performed by Shiadeh et al. (2019), which is a major warning sign of resistance to this broad-spectrum antibiotic. According to the WHO’s initial offices, American countries had the lowest rate of linezolid resistance in *Enterococcus faecalis* among all antibiotics. The Western Pacific, European, and American nations had the lowest prevalence of vancomycin resistance, while South-East Asia and the Eastern Mediterranean region had the highest prevalence. Additionally, it was discovered that Southeast Asia and America had the lowest and greatest levels of linezolid resistance for *Enterococcus faecium*, respectively. The highest rates of resistance were seen in Southeast Asian and Eastern Mediterranean nations, particularly for vancomycin and quinupristin/dalfopristin. Between 2000 and 2018, there were rising trends of antibiotic resistance in *Enterococcus* blood isolates, up to more than twice as much. Researchers concluded that these countries should be required to create acceptable rules for the use of antibiotics (Shiadeh et al., 2019) Asa1, hyl, cylA, efa, ebp, ace, esp., gelE, sprE, hemolysin, hydrogen peroxide, and biofilm production were among the virulence factors and antibiotic resistance that Gök et al. planned to examine in 2020 in *Enterococcus faecalis* and *E. faecalis* strains isolated from clinical specimens. The investigation contained a total of 110 *enterococcus* isolates that were recognised as infectious organisms. The isolates were identified, and virulence genes were found using the PCR technique. Of the 110 isolates of enterococci, 61 were determined to be *E. faecium* and 49 to be *E. faecalis*. Ciprofloxacin was shown to have the highest resistance rate (70.9%). Resistance to high level vancomycin and teicoplanin was 4.5%, high level gentamicin was 39.4%, high level streptomycin was 65.1%, and to ampicillin was 1.8% (Gök et al., 2020).

The level and distribution of resistance in commensal *E. faecalis* isolated from wild carnivorous mammals, as well as genetic linkages in terms of the source of these strains as well as resistance and virulence genes, were evaluated by Nowakiewicz et al. (2020). The findings revealed that over half (48%) of the examined animals carried one or more strains of *E. faecalis* that were resistant to multiple drugs. Moreover, two or three strains with distinct genotypes and resistance phenotypes were present in 44% of MDR-positive animals. High-level aminoglycoside resistance was present in a sizable proportion of bacteria (between 20% and as much as 57.5%) (Nowakiewicz et al., 2020). The percentage of antibiotic resistance profiles of *Enterococcus faecium*, *E. faecalis*, and *E. durans* isolated from traditional sheep and goat cheeses acquired from a chosen border region of Slovakia and Hungary (region Slanské vrchy) was calculated in Výrostková et al. (2021). *Enterococcus species*, including different strains of *E. faecalis, E. faecalis*, and *E. durans*, were found in cheese samples. *E. faecalis* strains were typically more resistant to antibiotics than *E. durans* and *E. faecium* strains. Their study showed high resistance to rifampicin (100%), vancomycin (85.7%), teicoplanin (71.4%), erythromycin (71.4%), minocycline (57.1%), nitrofurantoin (57.1%), ciprofloxacin (14.3%), and levofloxacin (14.3%) was seen in *E. faecalis*. Rifampicin (100%), teicoplanin (100%), vancomycin (66.7%), erythromycin (66.7%), nitrofurantoin (66.7%), and minocycline (33.3%) were all resistant to *E. durans*, while teicoplanin (100%), erythromycin (100%), and erythromycin (100%) were all resistant to *E. faecium* (Výrostková et al., 2021).

### 3.2 *Escherichia coli* and *Klebsiella pneumonia*

For the creation of medicines against MDR bacteria, the growth of extended-spectrum-lactamase (ESBL)-producing bacteria, especially *Escherichia coli* and *Klebsiella pneumoniae*, is of utmost importance (Chong et al., 2018). To evaluate the safety of bacterial isolates (*E. coli* strains) for human consumption as well as to give updated antibiotic data for effective patient treatment, Odonkor and Addo (2018) examined the microbiological quality and antibiograms of bacterial isolates from six different water sources in 2018. The results indicated that all the water sources tested had poor quality water. *E. coli*, *Enterobacter species*, *Klebsiella species*, *Salmonella typhi*, *Streptococcus species*, *Proteus vulgaris*, *Vibrio cholera*, *Shigella species*, *Pseudomonas aeruginosa*, and *Enterococcus faecalis* were among the bacteria that were isolated. MDR *E. coli* was present in 49.48% of cases. Isolates of *E. coli* exhibited high levels of antibiotic resistance. They were most resistant to tetracycline (21.45%), erythromycin (23.71%), cefuroxime (28.87%), and penicillin (31.9%). They were resistant to ciprofloxacin (74.2%), nalidixic acid (89.65%), nitrofurantoin (93.8%), cefotaxime and amikacin (91.75%), gentamicin (90.7%), chloramphenicol (69.07%), pipemidic acid (65.97%), and cefuroxime (52.58%) (Odonkor and Addo, 2018).

In a study published in Montso et al. (2019), isolated, identified, and characterised ESBL-producing *K. pneumoniae* and *E. coli* species from cattle faeces and samples of raw beef; Antimicrobial susceptibility testing showed that 66.7–100% of the isolates were multidrug-resistant and resistant to amoxicillin, aztreonam, ceftazidime, cefotaxime, and piperacillin. The ESBL gene determinants in the isolates that showed phenotypic resistance to ESBL antimicrobial susceptibility testing were checked and 53.1% of the isolates were found positive to have ESBL gene determinants (Montso et al., 2019).

After a thorough screening, Pormohammad et al. (2019) performed a meta-analysis on 39 studies, and the results showed that the overall MDR prevalence utilising the Disc Diffusion Method was 22, 31.3, and 5.7% in human, environmental, and animal *E. coli* isolates. MDR *E. coli* isolate rates were 12.6 and 22.2%, respectively, according to the results of the MIC. The incidence of MDR *E. coli* was higher in animal and environmental sources than in human sources, according to a comparison of isolates from various sources. Based on MIC, the prevalence of ESBL-producing *E. coli* was 42.4, 63.2, and 28.6%, respectively, in human, animal, and environmental or food isolates. Based on human, animal, and environmental/food isolates, the prevalence of ESBL-producing *E. coli* was 13, 26.3, and 25%, respectively. Animal isolates had a greater frequency of ESBL antibiotic resistance than observed in human isolates (Pormohammad et al., 2019).

For research on complex systems with multiple components, interactions and emergent processes are extremely important. Most of the research only considers pairwise interactions, ignoring higher-order interactions involving three or more components, Tekin et al. (2018) studied antibiotic combinations used to treat pathogenic *Escherichia coli* and gathered an unprecedented amount of detailed data (251 two-drug combinations, 1,512 three-drug combinations, 5,670 four-drug combinations, and 13,608 five-drug combinations). They discovered that the quantity of medications in the environment of the bacteria causes an increase in the frequency of higher-order interactions, which was directly contrary to earlier hypotheses and findings. More particularly, as more medications were introduced, rising incidences of emergent antagonistic effects (impact less than expected based on lower-order interaction effects), as well as increased occurrences of net synergy (effect more than expected based on independent individual effects), were observed. These results had significance for navigating issues related to the combinatorial complexity of multi-component systems and implications for the possible efficacy of medication combinations (Tekin et al., 2018). Nji et al. (2021) extracted study results obtained from 1989 to May 2020 in 2021. The search revealed a total of 9,363 publications, and 33 articles’ prevalence statistics were retrieved and combined. This revealed a pooled prevalence of antibiotic resistance (top ten antibiotics frequently prescribed in LMICs) in commensal *E. coli* isolates from human sources in community settings in LMICs, including ampicillin, cefotaxime, chloramphenicol, ciprofloxacin, co-trimoxazole, nalidixic acid, oxytetracycline, streptomycin, tetracycline and trimethoprim at 72% of 13,531 isolates, 27% of 6,700 isolates, 45% of 7,012 isolates, 7% of 10,618 isolates, 63% of 10,561 isolates, 30% of 9,819 isolates, 78% of 1,451 isolates, 58% of 3,831 isolates, 67% of 11,847 isolates and 67% of 3,265 isolates, respectively. The study evaluated the evidence of commensal *E. coli*’s high incidence of antibiotic resistance in community settings in low-middle-income countries in the study (Nji et al., 2021).

The ability of clinical *Klebsiella pneumonia* isolates from Egypt to produce biofilms was examined by Mohamed et al. (2020), along with the bacteria’s propensity for producing ESBLs, fimbrial genes, and antibiotic resistance. Ninety clinical *Klebsiella pneumonia* isolates were extracted from various sources in total. The determination of ESBL genotypic and phenotypic detection, biofilm assay, and antimicrobial susceptibility were done. The findings show a significant incidence of both biofilms forming capability (51%) and MDR (86.66%) among *Klebsiella pneumonia* isolates. In addition, *Klebsiella pneumonia* isolates that produced ESBLs were better able to form biofilms than those that did not. The prevalence of isolates with blaCTX-M was seen among ESBLS-biofilm makers, as were the occurrences of blaTEM and blaCTX-M. Fimbrial (mrkD and fimH) distribution among biofilm former isolates was 100 and 86.95%, respectively. ESBL-producing *Klebsiella pneumonia* isolates exhibited a higher potential to build a biofilm in contrast to non-ESBL-forming ones, according to the study, which also found that MDR was very prevalent among *Klebsiella pneumonia* in Egypt. Furthermore, our research provides compelling evidence that type 3 fimbriae greatly encourage biofilm development in *Klebsiella pneumonia* (Mohamed et al., 2020).

In Khaertynov et al. (2018) investigated the effects of antibiotic resistance and virulence variables in *K. pneumonia* strains on the clinical characteristics and outcomes of newborn infection. Testing of *K. pneumoniae* isolates for ESBL production in newborns with sepsis revealed positive results in 60% of cases, and in neonates with UTI—in 40% of cases. All *K. pneumoniae* isolates from blood and urine that produced ESBLs were resistant to third-generation cephalosporins and ampicillins. These isolates were additionally susceptible to meropenem, amikacin, and ciprofloxacin. Four blood isolates of *K. pneumoniae* and three urine isolates both contained the rmpA gene. In two cases, the rmpA gene was found in *K. pneumoniae* isolates that produce ESBL in neonates with sepsis. In two neonates with sepsis and three neonates with UTI, the genes for aerobactin and colibactin were found. Only rmpA-positive *K. pneumoniae* isolates were found in every case to have aerobactin and colibactin genes. We underestimate how often virulent *K. pneumoniae* strains are in newborns with sepsis and other neonatal infections. In this investigation, virulent strains of *K. pneumoniae* caused the most severe cases of newborn sepsis with a poor prognosis (Khaertynov et al., 2018).

The goal of the study by Gandor et al. (2022) was to characterise the Carbapenem-Resistant *K. pneumoniae* (CRKP) isolates collected from patients hospitalised in the ICU of the Zagazig University Hospitals (ZUHs) in Egypt. As for blaNDM, blaOXA-48, and blaKPC, respectively, about 56.2, 41.0, and 32.4% of the isolates showed the presence. Many isolates included genes that encoded carbapenemase, with blaNDM was the most present gene. When CRKP isolates were tested for antimicrobial susceptibility, an MDR profile was discovered, with 100% of the isolates being resistant to piperacillin, piperacillin/tazobactam, cefepime, ceftazidime, azithromycin, and ticarcillin. Contrarily, 95.0% of the isolates exhibited high levels of resistance to tobramycin, gentamicin (83.2%), pefloxacin (95%) and ciprofloxacin (98.3%) (Gandor et al., 2022). Amikacin (40.8%), aztreonam (73.3%), ceftazidime (75.7%), ciprofloxacin (59.8%), colistin (2.9%), cefotaxime (79.2%), cefepime (72.6%), and imipenem (65.6%) were the drugs with the highest prevalence rates of drug resistance in *K. pneumonia* studied by Effah et al. (2020). Some of the resistance-mediated genes identified were TEM (39.5%), SHV-11 (41.8%), and KPC-2 (14.6%). The most virulent components used by *K. pneumoniae* include the hyper-mucoviscous phenotype, genes associated with mucoviscosity, genes that are involved in the synthesis of lipopolysaccharide, genes involved in iron uptake and transport, and adhesive genes (Effah et al., 2020).

A fluorocycline belonging to the tetracycline class, eravacycline, was recently authorized. It is effective against a wide range of pathogenic infections, including *K. pneumoniae*, *Acinetobacter* spp., and *E. coli* that produces ESBLs (Yusuf et al., 2021). In Xu et al. (2022) showed that the clinical isolate Kp43 of the carbapenem-resistant *K. pneumoniae* rapidly established eravacycline resistance. The lon gene was disrupted by mutations or had its expression level decreased by a Tn insertion upstream of the lon gene, as per the WGS analysis of the resistant mutants. Eravacycline resistance in bacteria was decreased by complementation with a wild-type lon gene. Eravacycline-resistant strains that originated from a clinical isolate of *K. pneumoniae* that included NDM-1 were also found to have mutations in the lon gene (Xu et al., 2022). Both ceftazidime-avibactam and eravacycline are also vulnerable to Kp43. The US Food and Drug Administration (FDA) and the European Medicines Agency (EMA) separately approved ceftazidime-avibactam for use in clinical trials in 2015 and 2016. However, *K. pneumoniae* clinical isolates that were ceftazidime-avibactam resistant were reported in 2016 and afterwards (Shields et al., 2016, 2017; Galani et al., 2021).

### 3.3 Staphylococcus aureus

A condition that has been widely established in *Staphylococcus aureus* infections with various cephalosporins is that an organism’s *in vivo* susceptibility to a specific antibiotic may vary according to the size of the bacterial inoculum. There is evidence to show that some cephalosporins (such cefazolin) may not work in the case of high-inocula, deep-seated infections brought on by *S. aureus* that are susceptible to cephalosporins (Nannini et al., 2013).

With remarkable adaptive abilities, *Staphylococcus aureus* is a dangerous human pathogen. The primary mechanism through which antibiotic-resistant clones rapidly emerge is by acquiring antibiotic-resistance genes from other *S. aureus* strains or even from other genera (Haaber et al., 2017). Through a variety of processes, such as horizontal gene transfer and chromosomal mutation, resistance is rapidly evolving. *S. aureus* pathogenesis differs from common resistance mechanisms in that it can persist in the biofilm state on both surfaces that are biotic as well as abiotic. The World Health Organisation (WHO) identified MRSA as one of the top 12 infections that pose a hazard to human health (Craft et al., 2019). Many nosocomial infections are caused by *Staphylococcus aureus*. Nosocomial infections, also known as healthcare-associated infections (HAI), are infections or illnesses that develop while undergoing medical treatment but were absent at the time of admission. About 30% of people have *S. aureus*, a Gram-positive commensal, chronically colonising their skin and mucous membranes (Tong et al., 2015). Due to bacterial evolution and antibiotic misuse in recent years, *S. aureus* drug resistance has gradually grown, MRSA infection rates have climbed globally, and clinical anti-infective treatment for MRSA has gotten increasingly challenging (Guo et al., 2020).

Antibiotic resistance is also acquired by *S. aureus* from less closely related bacteria, including *enterococci* that exhibit vancomycin resistance (Haaber et al., 2017). The first MRSA strain with vanA and a minimum inhibitory concentration (MIC) to vancomycin greater than 1,000 mg/mL was discovered in 2002. A conjugative *Enterococcus faecalis* plasmid containing the vanA gene within the transposon element Tn1546 had been transmitted to an MRSA strain during coinfection, according to a genome study. The transposons then moved to a plasmid that was present in the original MRSA strain (Weigel et al., 2003). Since then, numerous instances of vancomycin-resistant *S. aureus* (VRSA) have been recorded; these cases frequently include the Tn1546 transposon element either being transferred by an enterococcalinc18-like plasmid or having transferred to a *S. aureus* plasmid (Haaber et al., 2017). According to Corvaglia et al. (2010) the transfer of genetic material from enterococci to *S. aureus* appears to be simpler in staphylococcal strains lacking a type-III endonuclease and the Hsd restriction modification systems. This transfer may also be facilitated by the presence of fp SK41 family plasmids in recipient *S. aureus* strains, possibly due to secreted plasmid factors (Zhu et al., 2013).

Antibiotic use in industrial hog operations (IHOs) can facilitate the growth of antibiotic-resistant (ABR) *Staphylococcus aureus*, was studied in 2017 by Hatcher et al. It’s yet unknown how much exposure to ABR *S. aureus* IHO employees and the kids who live in their houses have experienced. They looked at the prevalence of ABR *S. aureus* nasal carriage in individuals who had occupational exposure to IHOs versus those who did not, as well as in kids who lived in their homes. Individuals in IHO had a greater prevalence of *S. aureus* nasal carriage (53%) compared to Community Residents (31%) (adjusted prevalence ratio (aPR): 1.40), although MRSA nasal carriage was infrequent (2–3%) in both IHO and CR individuals. IHO employees and CR people both had a similar incidence of MDRSA nasal carriage (12% vs. 8%; aPR: 1.14). Nasal carriage prevalence of *S. aureus*, MRSA and MDRSA was greater among IHO children than CR children. Additionally, they discovered hints that suggested that children residing with IHO employees who reported taking personal protective equipment (PPE) home from the IHO had a greater incidence of *S. aureus*, MRSA, and MDRSA. Among IHO employees, *S. aureus* nasal carriage that was related to livestock predominated (Hatcher et al., 2017).

The peptidoglycan that forms when an MRSA strain is cultured with B-lactams is not well cross-linked. Exposure of the MRSA strain to β-lactams can lead to an increase in the pro-inflammatory effects of peptidoglycan, potentially contributing to pathology during infection (Müller et al., 2015). The global accessory gene regulator (Agr), which can be triggered in some MRSA strains, may not be able to do so due to the changed peptidoglycan structure (Rudkin et al., 2012). MRSA has become a barrier to clinical therapy due to its aspects of simple infection, high mortality, and antibiotic resistance (Khoshnood et al., 2019).

The prevalence of MRSA strains in *P. americana* and *B. germanica* cockroaches was 52.77 and 43.33%, respectively, according to a 2019 study by Abdolmaleki et al. MRSA strains were most prevalent (59.57%) in samples of *P. americana* cockroaches taken after external washing. The most common forms of resistance to penicillin (100%), ceftaroline (100%), tetracycline (100%), gentamicin (83.33%), and trimethoprim-sulfamethoxazole (80.55%) were found in MRSA isolates from external washing samples. The greatest rates of penicillin (100%), ceftaroline (100%), tetracycline (100%), trimethoprim-sulfamethoxazole (80%), and gentamicin (73.33%) resistance were seen in MRSA strains isolated from gut content samples (Abdolmaleki et al., 2019).

Gram-negative bacteria have a fundamentally different mechanism controlling entry into the persister stage than *S. aureus* (Conlon et al., 2016). *S. aureus* cells in stationary phase are resistant to bactericidal drugs, unlike Gram-negative bacteria. In any population of *S. aureus*, a tiny percentage of cells have already entered in the stationary phase, which is marked by ATP depletion (Foster, 2017). The entire population will enter stationary phase and turn into persisters if cells are purposefully deprived of ATP. A toxin-antitoxin system does not appear to be the mechanism(s) through which persister generation is accomplished. *S. aureus* cells in the persister state can be eliminated by the acylpepsipeptide antibiotic ADEP4 according to a study by Conlon et al. (2013). By releasing the enzyme from its ATP-dependent chaperone, it activates the ClpP protease, allowing it to uncontrollably break down intracellular proteins (Foster, 2017). Over 90% of *S. aureus* clinical isolates generate staphyloxanthin, which serves as a major virulence factor in the form of an antioxidant that protects against reactive oxygen species (ROS) produced by the host immune system (Liu et al., 2005, 2008). Therefore, preventing the manufacture of staphyloxanthin is a viable antivirulence tactic to stop *S. aureus* infection. The first beta-lactam antibiotic, penicillin, was shown to be a powerful tool against *S. aureus* infections in 1928. There were reports of *S. aureus* strains that were penicillin-resistant in the 1940s, just after it had been introduced into clinics (Rammelkamp and Maxon, 1942).

According to Gnanamani et al. (2017) the strains developed penicillinase, a plasmid-encoded beta-lactamase enzyme that enzymatically broke the beta-lactam ring of penicillin and rendered the antibiotic not active (Gnanamani et al., 2017). Penicillin resistance in the 1950s was only present in *S. aureus* hospital isolates. Due to plasmid transfer of the penicillinase gene (blaZ) and clonal spread of resistant strains, by the late 1960s, more than 80% of *S. aureus* isolates, regardless of their origin in the community or a hospital, were resistant to penicillin (Chambers, 2001; Lowy, 2003).

## 4 Novel antibiotics and AMR combating strategies

Most antibiotics currently in use come from the phylum *Actinobacteria*, with bacteria from the genus *Streptomyces* living in soil and producing over 80% of actinobacterial-derived antibiotics (Barka et al., 2016). The identification of new antibiotics designed to overcome resistance was used to address the growth of resistant strains of bacteria. This has gradually resulted in the selection of MDR bacteria, which have several resistance genes and are resistant to various types of antibiotics. A deeper understanding of the mechanisms and hotspots involved in the diffusion and development of novel resistances and MDR is urgently required considering their rapid emergence. Furthermore, resistance mechanisms aimed against newer antibiotics, such as-lactamases, frequently transmit resistance to earlier established antibiotics in the same class (Kakoullis et al., 2021). In terms of B-lactams, newly developed antibiotics comprise either novel cephalosporins or novel B-lactam inhibitors paired with existing B-lactams. Cephalosporins that have recently been developed include ceftobiprole, ceftaroline, cefiderocol, and ceftolozane, which is available in conjunction with tazobactam. Ceftobiprole is a fifth-generation cephalosporin and the first B-lactam to show antimicrobial efficacy against MRSA and VRSA *in vitro*. Ceftobiprole binds to PBP 2a, the altered PBP of MRSA, quickly and consistently. It is also effective against PBP 2x, a penicillin-resistant *Streptococcus pneumonia* PBP. Ceftobiprole, on the other hand, is sensitive to degradation by ESBLs, AmpC, and carbapenemases of Classes A, B, and D. Ceftobiprole is approved for the treatment of community and hospital-acquired pneumonia, as well as skin and soft-tissue infections such as diabetic foot infections (Lupia et al., 2021). Cefiderocol is a new siderophore cephalosporin with a structure identical to ceftazidime and cefepime but with a catechol moiety. Cefiderocol can bind to free iron because of catechol moiety, which mimics the naturally occurring siderophores of Gram-negative bacteria. Cefiderocol is actively carried into the periplasmic region after binding to iron, bypassing bacterial porins, where it dissociates from iron and binds to PBPs. Cefiderocol can thus accumulate in larger quantities in the periplasmic space than other cephalosporins. Cefiderocol is resistant to ESBLs, KPC (Class A), NDM, VIM, IMP (Class B), AmpC (Class C), and OXA-48 similar (Class D). It is permitted for the treatment of severe urinary tract infections, hospital-acquired pneumonia, and ventilator-associated pneumonia (Sato and Yamawaki, 2019; Abdul-Mutakabbir et al., 2020).

The World Health Organization’s recommended tuberculosis (TB) control method is known as directly observed therapy, short-course (DOTS, commonly known as TB-DOTS). According to WHO, “the most cost-effective way to stop the spread of tuberculosis in high-incidence communities is to cure it.” DOTS is the greatest cure for tuberculosis. DOTS includes five major components: (i) Establish a centralised and prioritised system for TB monitoring, documentation, and training. (ii) Detect cases by sputum smear microscopy. (iii) Standardised treatment regimen for six to 9 months, monitored by a healthcare provider or community health worker for the first 2 months. (iv) Drug supply. (v) Standardised recording and reporting system allowing treatment results assessment.

India established the Revised National Tuberculosis Control Programme (RNTCP) in 1997 to combat the growing TB epidemic, and it was expanded to encompass the whole country by 2006. This project investigated more than 650 TB suspects per 100,000 people, and over 1.5 million TB patients began the globally recommended Directly Observed Treatment Short-course (DOTS) per year. WHO created the DOTS approach for tuberculosis control in 1995, and it is presently being implemented in more than 180 countries worldwide. Under DOTS, TB mortality in India was lowered from 39 per 100,000 people in 1990 to 23 per 100,000 population in 2010, and the prevalence of TB was reduced to 256 per 100,000 population in 2010 from 456 per 100,000 population in 1997 (Chopra et al., 2018).

Ceftolozane is a new cephalosporin with a structure like ceftazidime that has been developed in conjunction with tazobactam. Ceftolozane-tazobactam’s range of effectiveness includes Gram-negative bacteria such as *P. aeruginosa*, *E. coli, K. pneumoniae, Burkholderia cepacia, and M. catarrhalis*. Ceftolozane is particularly effective against *P. aeruginosa* because it can evade many of the bacterium’s resistance mechanisms, such as the production of modified PBPs or the reduction of intracellular antibiotic concentrations via alterations in porins and the production of efflux pumps; it is even effective against *P. aeruginosa* strains resistant to ceftazidime or piperacillin-tazobactam. Ceftolozane-tazobactam has been authorised for the treatment of patients with complex intra-abdominal and urinary tract infections, and high-dosage ceftolozane-tazobactam is non-inferior to meropenem in the treatment of hospital acquired pneumonia (Sorbera et al., 2014; Zhanel et al., 2014; Kollef et al., 2019).

Omadacycline (aminomethylcycline subclass) and eravacycline (fluorocycline subclass) are two novel tetracyclines that are active against bacteria that contain efflux pumps or ribosome protection proteins, mechanisms that normally confer resistance to earlier tetracyclines (Koulenti et al., 2019; Shortridge et al., 2020; Eljaaly et al., 2021).

Dalbavancin, telavancin, and oritavancin are three new lipoglycopeptides used to treat MDR Gram-positive infections. Because of their various alterations, many antibiotics exhibit multiple modes of action in addition to inhibiting cell wall formation via transglycosylation and transpeptidation. The presence of lipid side chains in all three antibiotics allows them to attach to the cell membrane, significantly increasing their efficacy (Kakoullis et al., 2021).

Pharmacokinetics/pharmacodynamics-driven therapy has shown to be a viable strategy for optimising antimicrobial therapy based on the features of the specific patient being treated. It has become clear that human and veterinary pharmacologists share research goals of revising dosage regimens and developing drugs with improved pharmacokinetic/pharmacodynamic properties to maximise clinical efficacy, improve patient care, and reduce the adverse impacts of antimicrobial therapy on the naturally occurring microbiota (Guardabassi et al., 2020). In aquaculture, probiotics are used to fight both Gram-positive and Gram-negative bacteria. The application of Gram-positive bacteria as probiotics is common around the world. *Bacillus subtilis*, an endospore-forming bacillus genus, is widely used in aquaculture (Bhat and Altinok, 2023). To avoid such a dismal and oblique future, action must be taken now! Limiting the use of antimicrobials in farming and other phases of food production, as well as in medicine, can slow the spread of antibiotic resistance. Furthermore, further study is needed to discover and investigate other means of treating resistant diseases, such as the use of probiotics. Probiotics can be used to fight back on a bacterial level by replacing harmful germs with safer ones (Freeland et al., 2023). The World Bank’s recently released One Health (OH) operational framework prioritises AMR. The phrase ‘One Health’ refers to a collaborative, multi-sectoral, and transdisciplinary strategy that works at the local, regional, national, and global levels to achieve optimal health outcomes while acknowledging the interconnectedness of humans, animals, plants, and their common environment (Guardabassi et al., 2020).

Current One Health initiatives to AMR are mostly focused on reducing antibiotic usage in food animals. Recent systematic reviews and meta-analyses found associations between reduced antibiotic use in food animals and a decrease in AMR in animals, with limited evidence of a reduction in humans, recognising that the system’s complexity prevents the demonstration of a clear causal pathway and highlighting research gaps (White and Hughes, 2019).

Global changes are accelerating due to population growth, industrialization, and geopolitical issues, threatening biodiversity, ecosystems, and migratory patterns of both humans and other animals. Rapid climatic and environmental changes have resulted in the development and re-emergence of both infectious and non-infectious illnesses. Self-medication with antibiotics is an inappropriate approach to use antibiotics and a typical practice in resource-constrained nations that exposes people to the frightening danger of acquiring antimicrobial resistance and adverse effects (Torres et al., 2019). In India, the idea of One Health is still in its early stages, but it is gaining popularity. The Indian government has launched various measures to address emerging issues like as antibiotic resistance, zoonotic illnesses, and food safety with the OH approach, however there are numerous implementation hurdles (Aggarwal and Ramachandran, 2020) Antibiotic stewardship has evolved over the last 20 years and is now a worldwide initiative, with organisations striving to apply interventions to rationalise antibiotic usage in secondary care. Prescriber behaviours are influenced using frameworks from social science research. However, these frameworks frequently fail to integrate a contextual and cultural analysis (Charani and Holmes, 2019).

## 5 Influence of antibiotic resistance on sustainability

Antibiotics have saved millions of lives by lowering complications and death from infectious illnesses. However, increased antimicrobial medication usage is also linked to a rise in AMR. Pharmaceutical corporations, as manufacturers of these medications, can play a significant role in combating AMR. Without their efforts, the chances of successfully tackling the problem are slim. The rising use of antibiotics internationally, as well as the accompanying AMR, is increasing healthcare expenditures and adding to patient mortality.

A framework for analysis of AMR policies is a key step towards systematically consolidating information on previous and current policies, making sense of the diversity of national action against AMR, and relating this knowledge to nations’ AMR situations. Formulating AMR strategies that are tailored to the particular factors of AMR can help to provide an evidence-based and successful strategy for combating the worldwide problem of AMR (Ogyu et al., 2020). A Study was done by Huijbers et al. (2019) where they stated that Antibiotic resistance and environmental surveillance may help safeguard human, animal, and ecosystem health. However, the rationale for the selection of markers and sample locations that provide information about diverse risk situations is frequently missing. In the study, they described five fundamentally distinct goals for monitoring drugs and antibiotic resistance in the environment. The primary goal is to identify the risk of antibiotic-resistant microorganisms being transmitted to people through environmental channels. The second was which addresses the risk of speeding the evolution of antibiotic resistance in pathogens by contamination with agents and microbes of human or animal origin. The third goal was to address the risk that antibiotics represent for aquatic and terrestrial ecosystem health, particularly the effects on ecosystem functions and services. The other two objectives are similar to those of traditional clinical surveillance: to determine population-level resistance prevalence and population-level antibiotic usage. These two environmental monitoring aims are especially promising in areas where traditional clinical surveillance data and antibiotic usage statistics are few or unavailable. For each target, the levels of evidence offered by various phenotypic and genotypic microbial surveillance indicators, as well as antibiotic residues, were reviewed and evaluated conceptually. Furthermore, sites for which monitoring would be particularly useful are indicated. The suggested framework might be one of the beginning points for directing environmental monitoring and surveillance of antibiotics and antibiotic resistance at various spatiotemporal scales, as well as harmonising such operations with current human and animal surveillance (Huijbers et al., 2019).

India and China, the world’s largest makers of generic pharmaceuticals, are also top in antibiotic usage among developing countries. According to studies, antibiotic usage in India and China climbed by 63 and 65%, respectively, between 2000 and 2015 (Klein et al., 2018).

The UN announced the SDGs in 2015 as part of the “2030 Agenda for Sustainable Development” programme to serve as a worldwide blueprint for a better, more equal, and more sustainable living on our planet. The UN Earth Summit in Rio de Janeiro in 1992 and the UN Millennium Summit in New York in 2000 generated the precursors of SDGs & Agenda 21 and the Millennium Development Goals (MDGs) respectively. The SDG initiatives contain 17 well-defined objectives from the disciplines of environment, climate change, societal concerns, economics, education, and healthcare, all of which are frequently interconnected, as well as well-defined actions, targets, and monitoring criteria to enable the evaluation of these goals’ progress (Gajdács et al., 2021).

The growing levels of AMR pose a danger to the achievement of the SDGs since this phenomenon has a significant impact on changes in society and healthcare: the SDGs contextualise AMR as a global public health and social concern (World Health Organization, 2017). This may have been exacerbated by the onset of the global severe acute respiratory syndrome coronavirus 2 (SARS-CoV-2) pandemic, which has not only exacerbated societal inequalities and economic difficulties but has also resulted in a significant increase in antibiotic use worldwide for patient treatment; it is not yet known what the long-term consequences of this pandemic will be in the context of the SDGs (Hajek et al., 2020; Khor et al., 2020; Yadav, 2022; Yadav and Ravichandran, 2024).

The continued rise of AMR, for example, may hinder the achievement of Sustainable Development Goals 1 (No Poverty) and 2 (Zero Hunger). Food production is expected to increase by 50–70% between 2010 and 2030 due to increasing global population, economic growth, and changes in consumption habits; at the same time, the use of antimicrobials in food production (e.g., meat, milk, eggs) is expected to increase by a similar margin (Laxminarayan et al., 2016). Major reports have already demonstrated this: while global antibiotic consumption in human medicine increased by 10%, consumption of these drugs in animal husbandry increased by nearly 180% in the “BRICS” conglomerate countries (Brazil, Russia, India, China, and South Africa), with continued rising trends expected till 2030 which will lead to the spread of AMR across the ecosystem (Van Boeckel et al., 2017).

Several of the accepted objectives in the health-focused SDG 3 will be impossible to attain unless effective antibiotics are available. Antibiotics were included in a list of 13 life-saving commodities that contributed to the achievement of the Millennium Development Goals (MDGs) on maternal and child health in the Every Woman Every Child initiative. The advent of AMR is presently jeopardising the progress gained in lowering maternal mortality over the past 15 years, particularly in low-and middle-income countries (LMICs), where maternal death rates are up to 19 times higher than the rest of the world (Jasovský et al., 2016). Drug-resistant organisms have the potential to reverse the current good trend of reducing worldwide mortality rates from infectious illnesses, which have fallen from 23 to 17% of total fatalities in the preceding 15 years.

AMR impacts the poor, from the international development point of view. Resistance rates are high, and cheap treatment choices are frequently unavailable, especially since many patients pay for medications out of finances. Furthermore, poor sanitary systems facilitate disease transmission. Drug-resistant infections are more expensive, take longer to treat, and have a lower success rate than drug-susceptible illnesses. As a result of these considerations, AMR has a detrimental influence on national economic performance, potentially impeding progress towards SDG 1 (Laxminarayan et al., 2013).

Antibiotic residues and resistant bacteria are increasing in aquatic habitats as a result of waste products from hospitals, antibiotic production companies, and agriculture. Measurements at a wastewater treatment plant that received trash from around 90 pharma factories, for example, revealed that 45 kg of a popular antibiotic was dumped into a neighbouring river each day. The antibiotic concentration in the river was higher than it would be in a patient who had been treated with the medicine. As antibiotic residues are transported by water, sediments, and soil, gradients of differing antibiotic concentrations occur, and even very low antibiotic concentrations may be sufficient to select for highly resistant bacteria. Importantly, a lack of access to clean water and sanitation (SDG 6) promotes the spread of bacterial infections, resulting in increased morbidity and death, particularly among children (Jasovský et al., 2016).

Antimicrobial resistance to medicines currently causes an estimated 700,000 people each year and is expected to kill 10 million people and cost up to $100 trillion by 2050 if no effective action is taken. Loss of effective medicines for the treatment of infectious illnesses adds to state healthcare costs, lowers productivity, household income, and tax revenues, and leads to GDP losses. Such high expenses may jeopardise attempts to achieve long-term economic growth, as emphasised in SDG 8, making AMR a significant global development problem (Smith et al., 2005).

The recently introduced SDG indicator on antimicrobial resistance (AMR) intends to reduce the percentage of bloodstream infections caused by *Staphylococcus aureus* resistant to the drug methicillin (MRSA) and *Escherichia coli* resistant to 3rd-generation cephalosporin medicines. Controlling these two types of AMR pathogens effectively will eventually protect the ability to treat infections with current antimicrobials until new preventive and treatment strategies are developed. While providing data to monitor global trends, this new indicator will also be particularly useful for assessing progress in improving the country’s ability to successfully execute AMR and health security national action plans.

AMR is one of the issues that governments must deal with. Many people are unaware that they or their cattle may have a resistant illness, and those who treat them are frequently clueless as well. This suggests that the issue is mainly unidentified and unrecorded. The absence of representative AMR data, particularly in LMICs, remains an issue. Because the true scope of the problem and its potential consequences are unknown, developing a compelling national AMR narrative to boost political participation, financial commitments, and public awareness is challenging. The phase-out of the use of antimicrobial drugs for animal growth promotion and the implementation of pollution-control methods may need modifications in agricultural practices, waste and wastewater management, and pharmaceutical production systems. Controlling ecosystem-based ways to reduce antibiotic usage in plant production is critical for human health and the environment. These safeguards are a critical investment in the protection of human, animal, plant, and planetary health. Countries will thus require a combination of incentives, education, training, and legislation to effect these reforms (World Health Organization, 2021).

The link between AMR and increasing climate change is subtle, but significant, according to Goal 13 (Climate Action). The parallels between climate change and AMR are highlighted in the WEF report as creeping, multidisciplinary, and critical concerns for mankind (World Economic Forum, 2019). Changes in global climate will undoubtedly alter the diversity and complexity of microbial populations around the world; while the emergence of novel bacterial diseases is unlikely, the number of people at risk for many of these infections, including vector-borne and zoonotic pathogens, and illnesses associated with food, will undoubtedly increase (Turner, 2018; Fouladkhah et al., 2020). A 1°C rise in ambient temperatures may result in a 5–10% increase in the incidence of foodborne salmonellosis cases, resulting in significant healthcare and economic expenses. It has also been suggested that rising temperatures may facilitate the selection of resistant isolates.

## 6 Conclusion and future prospects

One of the crucial actions against prevailing antibiotic resistance is the identification of novel compounds to safeguard lives now and in the future. A significant strategy must be made in conjunction with determined efforts in the direction of infection prevention, better antibiotic use, and stopping the spread of resistance when it does arise (Centers for Disease Control and Prevention, 2019). Antibiotic misuse and overuse have led to a rise in bacterial resistance and a decline in susceptibility. Drugs and antibiotics are now less effective at treating bacterial infections and other diseases as a result. Drug concentrations at the infection site must both stop the organism and stay below levels hazardous to human cells. To boost effectiveness, new medication combinations targeting various targets and methods of action must be created. Antibiotic exposure has resulted in the development of resistance to every single agent used. Despite the difficulty of identifying novel antibiotics, the continual discovery of new antibiotics remains the major technique for treating bacterial-associated illnesses. While new antibiotics may be capable of combating existing resistance mechanisms, improper administration of these drugs will ultimately result in the development of resistance mechanisms against them as well. Additionally, new drugs must be created that target resistance mechanisms (for example, to target bacteria) by preventing the capacity of previous drugs to attack. Bacteria that are resistant to antibiotics are the root of a growing therapeutic issue that has detrimental effects on human health. There are major repercussions from the current global increase in bacterial strains that are resistant to antibiotics mixed with the downward trend in the creation of new antibiotics. Antimicrobial efficacy must be viewed as a finite global public benefit on the edge of becoming rare, and the world must work together to protect it to save many future victims of drug-resistant illnesses. It is required to have an adaptable, multifaceted strategy that works across the SDGs and involves a wide range of stakeholders. As a result, we cannot rely solely on the biomedical or health industries for solutions. AMR is a system breakdown that necessitates a multi-sectoral solution. By including AMR on the SDG agenda, we hope to strengthen and broaden worldwide political commitment to finding a solution to this developing issue.

## Author contributions

SS: Conceptualization, Data curation, Formal analysis, Investigation, Methodology, Writing – original draft, Writing – review & editing. AC: Conceptualization, Data curation, Formal analysis, Investigation, Methodology, Project administration, Supervision, Validation, Visualization, Writing – original draft, Writing – review & editing. AR: Conceptualization, Data curation, Formal analysis, Investigation, Methodology, Software, Supervision, Visualization, Writing – original draft, Writing – review & editing. DM: Conceptualization, Data curation, Formal analysis, Investigation, Visualization, Writing – original draft, Writing – review & editing. SH: Conceptualization, Data curation, Formal analysis, Investigation, Visualization, Writing – original draft, Writing – review & editing. SR: Data curation, Formal analysis, Investigation, Project administration, Visualization, Writing – original draft, Writing – review & editing. HT: Data curation, Formal analysis, Investigation, Methodology, Project administration, Supervision, Validation, Visualization, Writing – original draft, Writing – review & editing. TJ: Data curation, Formal analysis, Supervision, Validation, Writing – original draft, Writing – review & editing. VY: Formal analysis, Funding acquisition, Methodology, Project administration, Resources, Supervision, Validation, Visualization, Writing – original draft, Writing – review & editing.
